# Do Antimicrobial Peptides and Complement Collaborate in the Intestinal Mucosa?

**DOI:** 10.3389/fimmu.2015.00017

**Published:** 2015-01-30

**Authors:** Zoë A. Kopp, Umang Jain, Johan Van Limbergen, Andrew W. Stadnyk

**Affiliations:** ^1^Department of Microbiology and Immunology, Faculty of Medicine, Dalhousie University, Halifax, NS, Canada; ^2^Department of Pediatrics, Faculty of Medicine, Dalhousie University, Halifax, NS, Canada

**Keywords:** antimicrobial peptide, defensin, cathelicidin, Paneth cell, anaphylatoxin, lectin pathway, intestine, colitis

## Abstract

It is well understood that multiple antimicrobial peptides (AMPs) are constitutively deployed by the epithelium to bolster the innate defenses along the entire length of the intestines. In addition to this constitutive/homeostatic production, AMPs may be inducible and levels changed during disease. In contrast to this level of knowledge on AMP sources and roles in the intestines, our understanding of the complement cascade in the healthy and diseased intestines is rudimentary. Epithelial cells make many complement proteins and there is compelling evidence that complement becomes activated in the lumen. With the common goal of defending the host against microbes, the opportunities for cross-talk between these two processes is great, both in terms of actions on the target microbes but also on regulating the synthesis and secretion of the alternate family of molecules. This possibility is beginning to become apparent with the finding that colonic epithelial cells possess anaphylatoxin receptors. There still remains much to be learned about the possible points of collaboration between AMPs and complement, for example, whether there is reciprocal control over expression in the intestinal mucosa in homeostasis and restoring the balance following infection and inflammation.

## Intestinal Epithelium and Innate Defense

The cell boundary of the intestinal mucosa, interfacing the environment through the lumen, is comprised of a single-layer columnar epithelium, which in turn is composed of multiple cell types. These cells are undergoing constant renewal from epithelial stem cells in the crypt, with support of other epithelial and stromal cells in the niche. Progeny from the stem cells differentiate into the four specialized epithelial cell lineages; absorptive enterocytes with metabolic/digestive functions, mucus-secreting goblet cells, digestive-hormone secreting enteroendocrine cells, and Paneth cells. Paneth cells differ from other intestinal epithelial cells (IECs) in that they remain at the base of the crypts instead of migrating up the crypt during differentiation. Paneth cells are the major producer of antimicrobial peptides (AMPs) and they live longer than other IECs, surviving at the base of the crypt for approximately 20 days (1). IECs are interconnected through multiple molecular links but paramount among these are tight junctions, which control the permeability of the epithelial monolayer. Finally, scattered within the epithelium is a peculiar population of lymphocytes, the intraepithelial lymphocytes (IEL). IEL are squeezed between the basolateral borders of IECs and the two cell types communicate in maintaining the epithelial barrier (2). Mouse IEL reportedly express AMPs following exposure to bacteria (3). There undoubtedly remains more to be learned about IEL in defining the antimicrobial properties of the epithelium.

In addition to the cellular barrier, the innate defenses in the intestinal tract include highly glycosylated mucins (muc), secreted by goblet cells (4). The epithelium of the small intestine is overlayed with a single unattached mucus layer while two defined layers of mucus protect the colonic epithelium. In the colon, the inner layer is physically attached to the epithelium while the outer layer is unattached. Commensal microorganisms inhabit the outer, lower density mucus layer of the colon. Not surprisingly, degradation of the mucus layers permits contact between the IEC and bacteria. Illustrating the outcome of a comprised mucus layer, mice lacking MUC-2 develop colitis (5). In addition to mucins, goblet cells also produce trefoil factors, in particular trefoil factor 3 (TFF3), which facilitates mucin crosslinking and promotes epithelial repair, as well as resistin-like molecule-B (RELM-B), which stimulates MUC-2 secretion (4). TFF3 also induces a complement regulatory molecule, decay accelerating factor (DAF) on IEC (6). The mucus layer(s) are further impregnated with soluble factors that fortify the defensive capabilities. Secretory IgA, synthesized by B lymphocytes in the lamina propria, is transported into the mucus layer by IEC. Finally, AMPs and complement are found in the lumen, in the mucus.

## AMPs of the IEC

There are multiple families of AMPs suggesting an evolutionary divergence in the intestinal mucosa, a rich habitat for microbes and a principle route of infection of the host. AMPs are active against a variety of organisms including gram-positive and gram-negative bacteria, parasites, fungi, and enveloped viruses (Table 1) (7).

**Table 1:** **Properties of AMPs in the intestines**.

	Antimicrobial mechanism	Specificity	Murine version
**α-defensins**
HD-5	Pore-forming	Gram-negative, gram-positive, viruses, fungi, parasite	Cryptidins
HD-6	Nanonet	Gram-negative, gram-positive, viruses, fungi, protozoa	Cryptidins
**β-defensins**
hBD-1,2	Pore-forming	Gram-negative	mBD-1 (hBD-1) mBD-3 (hBD-2)
hBD-3	Pore-forming	Gram-negative, gram-positive	mBD-14
hBD-4	Pore-forming	Gram-negative, gram-positive, fungi	
**C-type lectin**
RegIIIα	Unknown	Gram-positive	RegIIIγ
**Cathelicidin**
LL-37	Pore-forming	Gram-positive, gram-negative, viruses, fungi, protozoa	CRAMP
**Others**
Secretory phospholipase A2	Degradation of membrane phospholipids	Gram-positive	–
Lysozyme	Peptidoglycan hydrolysis	Gram-positive	–

Although there are many AMPs, the majority share a few common structural features including an overall positive charge (due to lysine and arginine residues) and an increased attraction to the hydrophobicity of bacterial membranes, due to an abundance of hydrophobic amino acid residues (7). Mentioned earlier, Paneth cells are the main though not the exclusive source of AMPs. In response to IL-22, Toll-like receptor (TLR) and nucleotide oligomerization domain (NOD-2) signaling Paneth cells secrete lysozyme, secretory phospholipase A2 (sPLA2), α- and β-defensins, the C-type lectin regenerating islet-derived proteins (Reg), angiogenin 4, and cathelicidins in the small intestine, with the α-defensins being the most abundant (1, 4, 8). Paneth cell secretion of AMPs is important in maintaining spatial segregation of the intestinal microbiota from the epithelium (9). RegIIIγ-deficient mice consequently exhibit a defect in this segregation and microbes penetrate the mucus layer making intimate contact with host cells (10). In addition to providing AMPs, Paneth cells also help maintain crypt stem cells through the production of pro-growth factors such as WNT3 and Notch ligands (11).

Enterocytes are widely reported to produce AMPs including β-defensins (hBD-1,2,3,4), RegIIIα, and LL-37/human cationic AMP 18 (4, 12, 13). In fact, in mice temporary enterocyte expression of cathelin-related antimicrobial peptide (CRAMP) is important in allowing neonatal small intestinal colonization prior to the establishment of Paneth cells (14).

### Defensins

Defensins, arguably the most studied and well understood family of AMPs, target the surface membrane of microbes and function by forming pores leading to increased permeability of the membrane and the interruption of electrochemical gradients (Table 1) (15). The polypeptides are translated as an inactive precursor, which is cleaved to an active form. The primary protein sequence is a 87–94 residue peptide including a hydrophobic leader sequence, a short acidic pro-piece (which neutralizes the peptide), and a highly cationic mature sequence (7). Subtypes of defensins undergo different post-translational processing into an active cationic peptide, for example, in mice enteric α-defensins (cryptidins) are activated by matrix metalloproteinase matrilysin (MMP-7) (16). MMP-7 is a member of the metalloproteinase family of proteolytic enzymes produced by stromal fibroblasts and Paneth cells that degrade the extracellular matrix (17). Contrasting the situation in mouse cells, human Paneth cells only contain the pre-form of HD-5 and MMP-7 is undetectable (1). Thus, while human HD-5 was reported to be susceptible to MMP-7 cleavage, detection of a human homolog of the enzyme in the intestinal mucosa remains to be reported (18). Instead, an isoform of trypsin produced by Paneth cells was found to activate the protein resulting in multiple intermediates with variable levels of bactericidal activity (18, 19). Finally, a shorter amino-terminal extension in the human β-defensins permits bactericidal activity of the pre-forms (19). Otherwise the active defensins are 20–40 amino acids in length with three intramolecular disulfide bonds formed by a six-cysteine consensus sequence. The position of these intramolecular bonds is used to classify the family into α, β, and ζ defensins, with ζ defensins restricted to Rhesus monkeys (1).

#### α-Defensins

Alpha-defensins are classified based on a conserved pattern of six cysteines, which are linked 1–6, 2–4, and 3–5 (20). There are six subtypes of human α-defensins, four of which are found exclusively in neutrophils (HNP-1,2,3,4) and two of which are found in Paneth cells (HD-5,6), called the “enteric defensins” (1). Enteric defensins are found in rodents but the leukocyte α-defensins are not (1).

**Figure 1:**
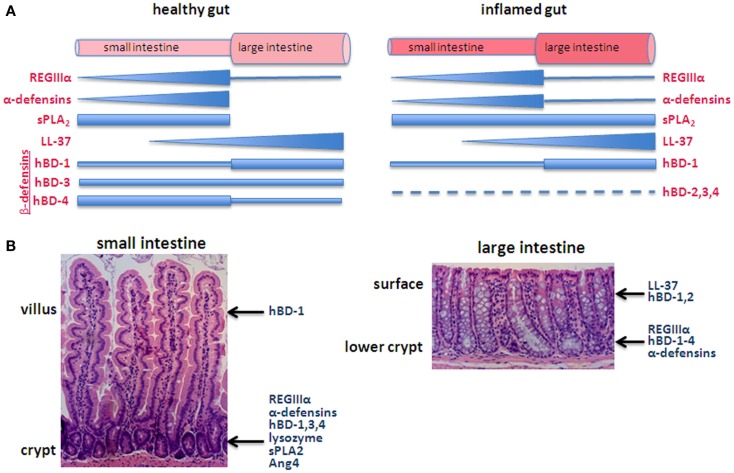
**Generalized depiction of AMP expression along the axes of the intestines**. **(A)** Pattern of expression in the longitudinal axis, comparing the healthy (left) with inflamed (right) intestines. The thickness of the bar/triangle for each AMP roughly depicts the relative concentration of that particular AMP. For example, RegIIIα is found along the small intestine with highest levels in the ileum and low levels in the large intestine. α-Defensins are also predominantly expressed in the small intestine with levels corresponding with the increasing abundance of Paneth cells from the duodenum to ileum. The longitudinal distribution of complement has not been characterized. **(B)** The epithelium of the small intestine is organized into crypts and villi, or in the case of the large intestine, crypts, and surface epithelium (e.g., lacks villi), which create a vertical axis along which differentiating cells migrate. Some AMPs are secreted from different cell types along this axis. For example, β-defensins are secreted by goblet cells, Paneth cells, and enterocytes and thus are produced in locations along the crypt-villus axis.

HD-5 exhibits direct bactericidal activity through a pore-forming mechanism but in an interesting contrast to the typical permeability-altering property of AMPs, HD-6 exhibits anti-bacterial activity indirectly (Table 1). HD-6 reportedly forms trap-like structures, which do not kill but instead immobilizes bacteria. HD-6 polymerizes to form peptide nanonets, which inhibit microbes from translocating across the intestinal barrier (21). Another difference is that HD-5 exhibits anti-parasite activity while HD-6 does not. Otherwise both human α-defensins share similar molecular structures, both exhibit anti-viral activity and both have been reported to be restricted to Paneth cells. HD-5 and HD-6 mRNAs are most highly concentrated in the ileum where Paneth cell abundance is highest (Figure 1A) (22). Relatively high levels of HD-5 are also detectable in the jejunum while levels of both HD-5 and 6 are low in the colon (21) (Figure 1).

#### β-Defensins

There are four human β-defensins (hBD-1,2,3,4), which are all expressed in keratinocytes and epithelial cells in a variety of human tissues (7, 20). The human β-defensins share a similar molecular structure with conserved cysteine residues linked 1–5, 2–4, 3–6 (20). With the exception of hBD-2, all human β-defensins are constitutively expressed in the small and large intestine (Figure 1). hBD-2 is unique in that it is detectable in low amounts in healthy tissues but is inducible with IL-1 (13). The molecules are expressed in Paneth cells and enterocytes and are all active against gram-negative bacteria. hBD-3 and hBD-4 additionally are active against gram-positive bacteria and are chemotactic for monocytes. hBD-1 mRNA is present in IEC at low levels throughout the intestines with highest expression in the colon (22).

#### RegIIIα

RegIIIα (also known as human hepatocarcinoma-intestine pancreas/pancreatitis-associated protein), is an AMP expressed in the liver, brain, and intestines of humans (23). It is present in the duodenum, jejunum, ileum, and colon, with expression highest in the crypts of the small intestine (Figure 1) (24). RegIIIα is a member of a large family of Reg genes but is one of only two RegIII genes found in humans (23). All are members of the C-type lectin family that bind glycan chains of peptidoglycan on the cell wall of gram-positive bacteria (25). The murine C-type lectin, RegIIIγ, is 65% identical to human RegIIIα and exhibits similar peptidoglycan binding (25). Paneth cells and enterocytes but not goblet cells express RegIIIγ (24, 25). Noteworthy, RegIIIγ lacks the complement recruitment domains present in other microbe-binding mammalian C-type lectins [such as mannose-binding lectin (MBL)] suggesting it is limited to direct anti-bactericidal activity (25).

#### LL-37

LL-37 is an AMP expressed in epithelial cells, keratinocytes, neutrophils, mast cells, monocytes, NK cells, B-cells, and γδ T cells (20). LL-37 belongs to the cathelicidin family of AMPs and functions in a similar way to defensins, by puncturing holes in the surface membrane of microbes (Table 1). LL-37 is unique in that in addition to antimicrobial activity, it has other immunological activities, acting through various receptors on cells (26). These activities include chemotaxis, wound healing, angiogenesis, degranulation of mast cells, and neutralizing lipopolysaccharide (LPS) and lymphotoxin-A (LTA) (26). LL-37 expression can be either constitutive or inducible. In the intestine, LL-37 is produced constitutively by cells above the transit-amplifying zone in colonic crypts, with lower levels detected in the small intestine (Figure 1B) (27). Production can be increased when these cells are stimulated with short chain fatty acids (27).

Thus a picture emerges in which the mucus layer resting on the healthy epithelium is rich with AMPs, although the pattern of expression along the intestines implies specializations among the different molecules (Figure 1) (11). The idea that AMPs show differential distribution is also evident at another level, roughly along the crypt-to-villus axis (Figure 1B). For example, β-defensins are secreted by goblet cells, Paneth cells, and enterocytes and thus are produced along the crypt-villus axis (22) while hBD-3,4 expression is highest in cells of the lower crypt (12). RegIIIα is produced by Paneth cells in the crypts of the small intestine and detected in the crypt and lower villi (24, 28). Similarly, α-defensins are predominantly expressed by Paneth cells in the base of the crypts although HD-5 has been detected in villous epithelial cells of the jejunum and ileum (13, 29).

### Epithelial AMP expression during inflammation

The cellular composition of the intestinal mucosa changes significantly during inflammation. This is in part due to the large numbers of infiltrating AMP-producing leukocytes as well as differences in the relative abundance of epithelial cell types resulting in altered expression of constitutive AMPs. One reported difference between the healthy and chronically inflamed intestinal epithelium impacting on AMP expression is an increased abundance of metaplastic Paneth cells in the colon (1). Colonic metaplastic Paneth cells produce α-defensins, lysozyme, and sPLA_2_ (1). sPLA_2_, in particular, is not detected in the healthy colon but is expressed by metaplastic Paneth cells as well as some colonocytes during inflammation (30). Other AMPs are also increased during inflammation. Murine RegIIIγ expression was increased during bacterial exposure and mucosal inflammation, and human RegIIIα expression was reported increased in patients with IBD (25, 31). Alpha-defensins are also induced in the large intestine during inflammation, associated again with metaplastic Paneth cells (1). Considering the importance of Paneth cells in providing AMPs, mutations in microbe sensing molecules in Paneth cells are thought to directly impact defensin production. However, the specific microbiome has emerged as an important factor in influencing defensin production even in mice with defects in these sensing molecules. This was demonstrated when defensin secretion by Nod2 gene knockout mice reverted to wild-type levels after exposure of the knockout mice to wild-type microbiota in co-housing experiments (32). Mixed findings have been reported for other AMPs and inflammation. hBD-1 levels have been reported to not change between the healthy and inflamed gut but contrarily, have also been shown to decline in ulcerative colitis (21, 33, 34). hBD-2,3, and 4 levels reportedly increase during ulcerative colitis but not Crohn’s disease (12). LL-37 expression reportedly does not change during inflammation (27, 35). Thus, the impact of inflammation on AMP expression varies based on the AMP and the specific disease, and the generalizations in Figure 1A should not be understood to apply to all diseases.

#### Studies from gene knockout mice

Much of what we know about the role of AMPs in the intestines is derived from research done using mice. The importance of α-defensins in gut homeostasis was shown by examining the gut microbes of matrilysin deficient (MAT^−/−^) mice, recalling that MMP-7 is required for α-defensin activation in mice (16). The lack of active α-defensins resulted in an impaired ability to control levels of both non-invasive and invasive bacteria in the intestines (16). Additionally, the oral lethal dose of an invasive strain of *S. typhimurium* was 90% less than that of the wild-type mice (16). Similar findings of increased susceptibility to bacteria were observed with mice experiencing graft versus host disease, which includes injury to Paneth cells resulting in reduced α-defensin production. Reduced α-defensin expression in turn was associated with changes in commensal bacteria populations and lower numbers of the major enteric commensals and higher numbers of minor enteric commensals (*E. coli*) led to septicemia in the mice (36).

Alpha-defensin expression in mice has been reported to be Nod2 dependent and consequently the Nod2 gene knockout mouse (Nod2^−/−^) has been a popular model to study (37). Nod2^−/−^ mice reportedly have higher levels of commensal bacteria as well as a reduced ability to prevent pathogenic enteric bacterial colonization (38). Nod2^−/−^ mice infected by *L. monocytogenes* had lower numbers of specific cryptidins in their terminal ileum and were less successful in defending against the pathogen than wild-type mice. The mice also had larger populations of bacteria in their livers and spleens (37). Nod2^−/−^ mice challenged with *Helicobacter hepaticus* suffered from granulomatous inflammation of the ileum but Nod2^−/−^ expressing transgenic HD-5 killed the bacteria (39). However, again, these experiments were conducted without necessarily controlling for the impact of the microbiota.

Transgenic expression of HD-5 was used in other infectious models. Compared to wild-type mice, overexpression of HD-5 protected mice from infection by *S. typhimurium*. Wild-type strain mice died while the transgenic mice experienced less severe disease, less colonization, and recovered (40). These outcomes support the idea that defensins mediate protection beyond the regulation of commensal populations of microorganisms.

The role of cathelicidins has also been explored using mouse models. In addition to the role of CRAMP in colonization of the neonatal gut, CRAMP is also involved in the response to injury. Mouse colons inflamed with DSS showed increased levels of mCRAMP. mCRAMP^−/−^ mice experienced worse colitis, which was reversible using exogenous mCRAMP or mCRAMP-encoding plasmids, confirming that the cathelicidins are protective in the colon (41, 42). In another study, cathelicidin deficient mice (camp^−/−^) displayed a thinner inner colonic mucus layer than wild-type mice and had lesions on the surface epithelium due to a higher incidence of penetration and colonization by *E. coli O157* (43).

Similar to the camp^−/−^ mice, RegIIIγ^−/−^ mice also presented with changes in mucus distribution and incidence of bacteria on the mucosa of the ileum (44). A significant reduction in the amount of mucus was detected in RegIIIγ^−/−^ mice due to changes in MUC-2 expression (44). Bacteria were observed in contact with the surface epithelium in the knockout mice; however, it is unclear whether this was due to the absence of the bactericidal activity of RegIIIγ or a consequence of changes in mucus distribution. RegIIIγ^−/−^ mice were also reported to have higher numbers of gram-negative bacteria in their feces (44).

Taken together, the evidence that AMPs are important in the defense of the healthy and inflamed intestines is compelling. Yet it is not entirely clear why there is such a diversity of AMPs, some with varying patterns of expression along the length of the intestines. Additionally, even in the studies using gene knockout mice, it cannot be concluded that the AMPs act alone and directly to affect the phenotypes reported (for example, the role of the microbiome in shaping the AMP response was illustrated in Nod2^−/−^ mice). There remains a high likelihood that AMPs act in concert with other defenses to achieve homeostasis and recover following injury and inflammation. The complement system is now emerging as one such parallel defense mechanism.

## Complement and the Intestine

Complement comprises a set of soluble proteins and membrane receptors and regulators that function in a highly coordinated manner to destroy microbes and facilitate removal of apoptotic/necrotic cells. Split complement molecules link the innate and adaptive immune systems, indirectly by acting on antigen presenting cells and directly by acting on leukocytes including lymphocytes. Despite the known crucial involvement of these functions in modulating the local response to microbes, the role of complement in the intestines is not completely understood.

Complement activation primarily occurs through one of the three pathways: the classical pathway (CP), lectin pathway (LP), and/or alternative pathway (AP) – all converging at the C3 convertase step. C3 convertases cleave C3 into C3a and C3b. C3b then associates with the C3 convertase to form a C5 convertase, which cleaves C5 into C5a and C5b. C5b become the nidus for binding C6, C7, C8, and C9 molecules to form the membrane attack complex (MAC), the lytic machinery of complement.

Each route of activation has proximal effectors that double as pattern recognition molecules. C1q, a proximal CP protein, combines with immune complexes forms a multimolecular complex with serine proteases, C1r and C1s. This complex cleaves C4 then associates with the product C4b into a complex, which cleaves C2 to form the CP C3 convertase. The LP is initiated by binding of MBL/ficolins to the mannose residues on microbial surfaces. Bound MBL recruits MBL-associated serine proteases (MASP-1 and MASP-2) that function similar to C1r and C1s by cleaving C4 and C2 to form the classical C3 convertase. The AP is unique as it does not require pattern recognition molecules to become activated. Instead, a “tick over” mechanism involves the spontaneous hydrolysis of C3 into C3(H_2_O), which behaves similar to C3b and binds factor B (fB). Through a series of reactions involving factor D (fD) and properdin, the C3bBb complex forms the AP C3 convertase [reviewed in Ref. (45)]. Two additional models of AP activation have been proposed; (1) properdin, acting as a pattern recognition molecule, binds to a surface and provides a platform for C3 convertase assembly and, (2) C3b attached to a surface binds properdin, which in turn promotes AP convertase formation (46–48). In addition to the three principal pathways, evidence has emerged showing that complement may be activated through other mechanisms. For example, MBL can cleave C3 through a C2 by-pass activation mechanism (49, 50). MASPs reportedly cleave C3 to C3b thereby triggering the AP (51). Additionally, MASP-1, without the requirement of MBL, may cleave factor D from the pro- to mature form, again leading to AP activation (52). Finally, some coagulation pathway proteases can directly cleave C5 and C3 (53). Finally, an opinion has emerged that the AP may not be an entirely independent pathway but rather is responsible for complement amplification that was initiated by other pathways (54, 55).

### Complement in the intestinal lumen

With regard to bolstering innate defenses in the mucosa, it is important to know whether complement proteins are present in the lumen. This question has not been systematically or comprehensively studied and the current understanding is incomplete. In support of complement in the lumen are published accounts of split complement molecules on the mucosa. In one example, C3b and MAC proteins were detected on the surface epithelium in patients with Crohn’s disease and ulcerative colitis (56, 57). In another report that measured complement in lumen samples, higher C3 and C4 levels were found in jejunal secretions from Crohn’s disease patients compared to healthy participants (58). Complement proteins were also detected in lumen samples collected from the small intestine of patients experiencing bacterial overgrowth (59). Noteworthy, and at odds with complement in the lumen, these authors failed to find MAC proteins, suggesting the MAC is not present (active) in the lumen. When considering where complement in the lumen may be derived from the pancreatic epithelium has been identified as a source of complement, with exocrine secretions arming at least the duodenum (60). Otherwise multiple reports identify epithelial cells as a source, for example, C4 mRNA was detected in both healthy participants’ and Crohn’s disease patients’ mucosa while C3 expression was limited to crypt cells in inflamed samples (61). A lack of C3 detection in epithelial cells was repeated in another study that did detect C3 in colonic subepithelial myofibroblasts (62). Factor B proved to be among the most highly increased complement molecules in epithelial cells in inflamed mucosa of IBD patients compared to healthy donor mucosa, suggesting that inflammation leads to arming the AP in the intestine (63). On the other hand, MBL has not been detected in mouse or human mucosa suggesting this proximal activator of the LP may not be active (64). Despite the lack of C3 detection using *in situ* techniques, C3 has commonly been found in epithelial cell lines. Bemet-Camard et al. reported that T84, Caco-2, HT-29, and a non-transformed IEC line (INT407) were all positive for C3 and C4 (65). Another study repeated the finding that Caco-2 cells produce C4 and fB but also C3 (66). In addition to constitutive production, stimulation of IEC with cytokines such as TNF, IL-6, and IL-1β increased the production of complement proteins (66). However, consistent with the lack of MAC in the luminal secretions from small intestine, this study failed to detect MAC mRNAs. How is the lack of local MAC synthesis compatible with the detection of MAC on the mucosa? Bleeding in ulcerated parts of the intestines could result in complement in the lumen. Additionally, infiltrating leukocytes make complement and could act as a source. Neutrophils are the leading source of properdin and by providing properdin following infiltration these cells, neutrophils may mediate AP amplification in the lumen (46, 67). Altogether, these findings suggest that the intestinal epithelium does not synthesize all the complement molecules and this raises questions over what role complement therefore plays in the healthy intestines. During inflammation, the remaining proteins are perhaps provided by infiltrating cells and/or blood resulting in complete pathways, and local activation becomes possible. Finally, the idea that complement activation occurs, including through the MAC, is indirectly supported by epithelial cells possessing CD55 and CD59 on the apical surface. These membrane proteins are negative regulators of the convertases and MAC, respectively (68). As for complement impacting the intestinal epithelium from the lumen, we reported that cell lines apically express the C5aR and respond to C5a with increased CXCL8 mRNA, introducing the possibility that anaphylatoxins provide danger signals to the epithelium (69). In summary, many complement proteins are present in the uninflamed mucosa and complement is activated during inflammatory conditions, opening the possibility that AMPs and complement may collaborate to enhance the innate defenses in health and disease in the intestines.

## Complement and AMP Cross-Talk at the Intestinal Mucosa

**Figure 2:**
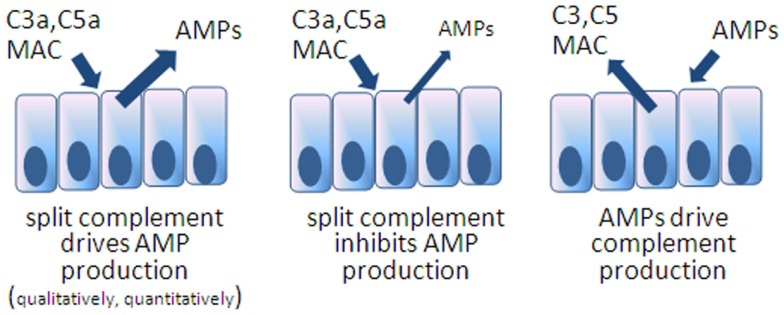
**Models speculating on reciprocal interactions between AMPs and complement expression in the intestines**. The first model is inferred from the example of C5aR blocked mice showing deficiencies in skin AMPs. The others remain to be tested.

Secreted into the mucus layer, there is good reason to suppose these two antimicrobial systems interact on microbial targets but also possibly through the reciprocal regulation of expression, whether agonistically and/or antagonistically (Figure 2). The fact is that such interactions have not yet been described in the intestines and we can only hypothesize on the manner of interaction. Noteworthy is a recent report from Chehoud et al. who found that antagonizing C5aR resulted in a decline in diversity of the skin microbiota of healthy mice associated with changes in immune effectors including AMPs (70). Such a relationship between split complement effectors, AMPs, and microbes could certainly be active in the intestines.

### AMP/complement collaboration?

One obvious functional convergence between AMPs and complement is the common antimicrobial activity. Complement activation leads to antimicrobial activity with the MAC but surprisingly, C3a also has microbicidal activity. The antimicrobial specificity of C3a and other peptides following cleavage of C3 is broad, including gram-negative and gram-positive bacteria (71) and fungi (72). C3 has a long history of evolution and was present before elements of the MAC evolved. Perhaps primitive organisms depended on C3 split products having direct antimicrobial activities. Perhaps also related to this long evolutionary history is the discovery that cells, including epithelial cells, constitutively make and cleave C3 into C3a as an autocrine survival signal (73). It will be interesting to know whether there is a relationship between C3a antimicrobial potency and organisms which depend more on innate defenses, compared to higher vertebrates with adaptive immune defenses.

In addition to acting independently to repel microbes, these two systems may collaborate by reducing the effective concentration needed for lysis when both are present. Experiments to test this potential have not yet been reported but are certainly achievable. In another manner, HD-6 presents an interesting possibility for collaboration with complement, drawing on an example from neutrophils. Mentioned earlier, HD-6 forms a lattice in the mucus; how closely this lattice resembles neutrophil extracellular traps (NETs) is not clear but NETs do collaborate with complement. NETs are the discharged nuclear and cytoplasm contents of dying neutrophils giving their best final effort to impede microbes. C1q binds to NETs leading to complement activation (74). The HD-6 lattice could function as a foundation for focusing complement activation by complement pattern recognition molecules like properdin. By this mechanism the contribution of HD-6 may be ensuring complement activation occurs, in addition to the physical impedance of microbes.

Contradicting the speculation that the two systems may act in synergy is a report showing that an AMP can interrupt complement activation, at least *in vitro*. Bhat and co-workers found that HBD-2 (but not HBD-1) bound C1q and prevented activation of the classical pathway *in vitro* (75).

### Reciprocal control over expression

Another likely point of interaction between complement and AMPs is the possibility that there is reciprocal control over expression of the two systems (Figure 2). An example can be drawn from more primitive organisms, mosquitoes, where C3-like molecules drive AMP expression in order to control viruses (76). In this regard, it would be interesting to know whether mice deficient in specific AMPs, as discussed earlier, respond by increasing local complement concentrations or possibly respond with reduced levels if the AMP directly impact complement synthesis. On the other hand, and considering that some AMPs are constitutively produced and that complement is present but not activated (with perhaps cytoplasmic C3 being the exception), the reciprocal situation may be true: that activated complement products impact AMP expression. Anaphylatoxin receptor gene knockout mice have been applied in various models of colitis with an incomplete understanding of whether other innate defenses have been compromised. A rational line of investigation will be to determine the impact of anaphylatoxins on AMP expression in IEC, including Paneth cells.

## Conclusion

Considerable attention and progress has been made in understanding the cell sources and contribution of AMPs to defense of the intestines. Multiple AMPs show regional patterns of expression in both axes of the intestines, longitudinally and within the crypt-to-villus axis. The other antimicrobial system, complement, is present and becomes activated in the mucosa but we are only beginning to explore the impact of this activation, including on AMP activity and expression. We offer some hypotheses for further investigation into what is certain to be a closely coordinated collaboration between the two systems in the defense of the host.

## Author Contributions

All authors researched the content, composed a section of the review, edited multiple drafts, and gave final approval of the manuscript.

## Conflict of Interest Statement

The authors declare that the research was conducted in the absence of any commercial or financial relationships that could be construed as a potential conflict of interest.

## References

[1] Cunliffe RN Mahida YR. Expression and regulation of antimicrobial peptides in the gastrointestinal tract. J Leukoc Biol (2004) 75:49–58. 10.1189/jlb.0503249 14525966

[2] Inagaki-Ohara K Dewi FN Hisaeda H Smith AL Jimi F Miyahira M Intestinal intraepithelial lymphocytes sustain the epithelial barrier function against *Eimeria vermiformis* infection. Infect Immun (2006) 74:5292–301. 10.1128/IAI.02024-05 16926423 PMC1594832

[3] Ismail AS Severson KM Vaishnava S Behrendt CL Yu X Benjamin JL Gammadelta intraepithelial lymphocytes are essential mediators of host-microbial homeostasis at the intestinal mucosal surface. Proc Natl Acad Sci U S A (2011) 108:8743–8. 10.1073/pnas.1019574108 21555560 PMC3102410

[4] Peterson LW Artis D. Intestinal epithelial cells: regulators of barrier function and immune homeostasis. Nat Rev Immunol (2014) 14:141–52. 10.1038/nri3608 24566914

[5] Wenzel UA Magnusson MK Rydström A Jonstrand C Hengst J Johansson MEV Spontaneous colitis in Muc2-deficient mice reflects clinical and cellular features of active ulcerative colitis. PLoS One (2014) 9:e100217. 10.1371/journal.pone.0100217 24945909 PMC4063762

[6] Andoh A Kinoshita K Rosenberg I Podolsky DK. Intestinal trefoil factor induces decay-accelerating factor expression and enhances the protective activities against complement activation in intestinal epithelial cells. J Immunol (2001) 167:3887–93. 10.4049/jimmunol.167.7.3887 11564806

[7] Kaiser V Diamond G Expression of mammalian defensin genes. J Leukoc Biol (2000) 68:779–84. 11129644

[8] Clevers HC Bevins CL. Paneth cells: maestros of the small intestinal crypts. Annu Rev Physiol (2013) 75:289–311. 10.1146/annurev-physiol-030212-183744 23398152

[9] Chu H Mazmanian SK. Innate immune recognition of the microbiota promotes host-microbial symbiosis. Nat Immunol (2013) 14:668–75. 10.1038/ni.2635 23778794 PMC4109969

[10] Vaishnava S Yamamoto M Severson KM Ruhn KA Yu X Koren O The antibacterial lectin RegIIIgamma promotes the spatial segregation of microbiota and host in the intestine. Science (2011) 334:255–8. 10.1126/science.1209791 21998396 PMC3321924

[11] Mowat AM Agace WW. Regional specialization within the intestinal immune system. Nat Rev Immunol (2014) 14:667–85. 10.1038/nri3738 25234148

[12] Fahlgren A Hammarström S Danielsson Å Hammarström M-L. Beta-defensin-3 and -4 in intestinal epithelial cells display increased mRNA expression in ulcerative colitis. Clin Exp Immunol (2004) 137:379–85. 10.1111/j.1365-2249.2004.02543.x 15270856 PMC1809118

[13] O’Neil DA Porter EM Elewaut D Anderson GM Eckmann L Ganz T Expression and regulation of the human β-defensins hBD-1 and hBD-2 in intestinal epithelium. J Immunol (1999) 163:6718–24. 10586069

[14] Ménard S Förster V Lotz M Gütle D Duerr CU Gallo RL Developmental switch of intestinal antimicrobial peptide expression. J Exp Med (2008) 205:183–93. 10.1084/jem.20071022 18180308 PMC2234368

[15] Ouellette A Bevins CL Paneth cell defensins and innate immunity of the small intestine. Inflamm Bowel Dis (2001) 7:43–50 10.1097/00054725-200102000-00007 11233660

[16] Wilson CL Ouellette AJ Stachell DP Ayabe T López-Boado YS Stratman JL Regulation of intestinal α-defensin activation by the metalloproteinase matrilysin in innate host defense. Science (1999) 286:113–7. 10.1126/science.286.5437.113 10506557

[17] Collins HM Morris TM Watson SA. Spectrum of matrix metalloproteinase expression in primary and metastatic colon cancer: relationship to the tissue inhibitors of metalloproteinases and membrane type-1-matrix matalloproteinase. Br J Cancer (2001) 84:1664–70. 10.1054/bjoc.2001.1831 11401321 PMC2363688

[18] Ghosh D Porter E Shen B Lee SK Wilk D Drazba J Paneth cell trypsin is the processing enzyme for human defensin-5. Nat Immunol (2002) 3:583–90. 10.1038/ni797 12021776

[19] Wilson CL Schmidt AP Pirila E Valcore EV Ferri N Sorsa T Differential processing of α- and β-defensin precursors by matrix metalloproteinase-7 (MMP-7). J Biol Chem (2009) 284:8301–11. 10.1074/jbc.M809744200 19181662 PMC2659188

[20] Yang D Biragyn A Hoover DM Lubkowski J Oppenheim JJ. Multiple roles of antimicrobial defensins, cathelicidins, and eosinophil-derived neurotoxin in host defense. Annu Rev Immunol (2004) 22:181–215. 10.1146/annurev.immunol.22.012703.104603 15032578

[21] Ostaff MJ Stange EF Wehkamp J. Antimicrobial peptides and gut microbiota in homeostasis and pathology. EMBO Mol Med (2013) 5:1465–83. 10.1002/emmm.201201773 24039130 PMC3799574

[22] Frye M Bargon J Lembcke B Wagner TOF Gropp R. Differential expression of human α- and β-defensins mRNA in gastrointestinal epithelia. Eur J Clin Invest (2000) 30:695–701. 10.1046/j.1365-2362.2000.00696.x 10964161

[23] Nata K Liu Y Xu L Ikeda T Akiyama T Noguchi N Molecular cloning, expression and chromosomal localization of a novel human REG family gene, REG III. Gene (2004) 340:161–70. 10.1016/j.gene.2004.06.010 15556304

[24] Matsumoto S Konishi H Maeda R Kiryu-Seo S Kiyama H. Expression analysis of the regenerating gene (Reg) family members Reg-IIIβ and Reg-IIIγ in the mouse during development. J Comp Neurol (2012) 520:479–94. 10.1002/cne.22705 21681751

[25] Cash HL Whitham CV Behrendt CL Hooper LV. Symbiotic bacteria direct expression of an intestinal bactericidal lectin. Science (2006) 313:1126–30. 10.1126/science.1127119 16931762 PMC2716667

[26] Vandamme D Landuyt B Luyten W Schoofs L. A comprehensive summary of LL-37, the factotum human cathelicidin peptide. Cell Immunol (2012) 280:22–36. 10.1016/j.cellimm.2012.11.009 23246832

[27] Hase K Eckmann L Leopard JD Varki N Kagnoff MF. Cell differentiation is a key determinant of cathelicidin LL-37/human cationic antimicrobial protein 18 expression by human colon epithelium. Infect Immun (2002) 70:953–63. 10.1128/IAI.70.2.953-963.2002 11796631 PMC127717

[28] Ogawa H Fukushima K Naito H Funayama Y Unno M Takahashi K Increased expression of *HIP/PAP* and *regenerating gene III* in human inflammatory bowel disease and a murine bacterial reconstitution model. Inflamm Bowel Dis (2003) 9:162–70. 10.1097/00054725-200305000-00003 12792221

[29] Cunliffe RN Rose FRAJ Keyte J Abberley L Chan WC Mahida YR. Human defensin 5 is stored in precursor form in normal Paneth cells and is expressed by some villous epithelial cells and by metaplastic Paneth cell in the colon in inflammatory bowel disease. Gut (2001) 48:176–85. 10.1136/gut.48.2.176 11156637 PMC1728187

[30] Haapamäki MM Grönroos JM Nurmi H Alanen K Kallajoki M Nevalainen TJ. Gene expression of group II phospholipase A2 in intestine in ulcerative colitis. Gut (1997) 40:95–101. 10.1136/gut.40.1.95 9155583 PMC1027015

[31] Shi J. Defensins and Paneth cells in inflammatory bowel disease. Inflamm Bowel Dis (2007) 13:1284–92. 10.1002/ibd.20197 17567878

[32] Shanahan MT Carroll IM Grossniklaus E White A von Furstenberg RJ Barner R Mouse Paneth cell antimicrobial function is independent of Nod2. Gut (2014) 63:903–10. 10.1136/gutjnl-2012-304190 23512834 PMC3844066

[33] Fahlgren A Hammarström S Danielsson Å Hammarström M-L. Increased expression of antimicrobial peptides and lysozyme in colonic cells of patients with ulcerative colitis. Clin Exp Immunol (2003) 131:90–101. 10.1046/j.1365-2249.2003.02035.x 12519391 PMC1808590

[34] Natividad JMM Hayes CL Motta J-P Jury J Galipeau HJ Philip V Differential induction of antimicrobial REGIII by the intestinal microbiota and *Bifidobacterium breve* NCC2950. Appl Environ Microbiol (2013) 79:7745–54. 10.1128/AEM.02470-13 24096422 PMC3837813

[35] Schauber J Svanholm C Termén S Iffland K Menzel T Scheppach W Expression of the cathelicidin LL-37 is modulated by short chain fatty acids in colonocytes: relevance of signalling pathways. Gut (2003) 52:735–41. 10.1136/gut.52.5.735 12692061 PMC1773650

[36] Eriguchi Y Takashima S Oka H Shimoji S Nakamura K Uryu H Graft-versus host disease disrupts intestinal microbial ecology by inhibiting Paneth cell production of α-defensins. Blood (2012) 120:223–31. 10.1182/blood-2011-12-401166 22535662

[37] Kobayashi KS Chamaillard M Ogura Y Henegariu O Inohara N Nuñez G Nod-2-dependent regulation of innate and adaptive immunity in the intestinal tract. Science (2005) 307:731–4. 10.1126/science.1104911 15692051

[38] Petnicki-Ocwieja T Nrncir T Liu Y-J Biswas A Hudcovic T Tlaskalova-Hogenova H Nod2 is required for the regulation of commensal microbiota in the intestine. Proc Natl Acad Sci U S A (2009) 106:15813–8. 10.1073/pnas.0907722106 19805227 PMC2747201

[39] Biswas A Liu Y-J Hao L Mizoguchi A Salzman NH Bevins CL Induction and rescue of Nod2-dependent Th1-driven granulomatous inflammation of the ileum. Proc Natl Acad Sci U S A (2010) 107:14739–44. 10.1073/pnas.1003363107 20679225 PMC2930434

[40] Salzman NH Ghosh D Huttner KM Paterson Y Bevins CL. Protection against enteric salmonellosis in transgenic mice expressing a human intestinal defensin. Nature (2003) 422:522–6. 10.1038/nature01520 12660734

[41] Koon HW Shih DQ Chen J Bakirtzi K Hing TC Law I Cathelicidin signaling via the toll-like receptor protects against colitis in mice. Gastroenterology (2011) 141:1852–63. 10.1053/j.gastro.2011.06.079 21762664 PMC3199285

[42] Tai E Wu W Wang X Wong HPS Yu L Li ZJ Intrarectal administration of mCRAMP-encoding plasmid reverses exacerbated colitis in Cnlp^-/-^ mice. Gene Ther (2013) 20:187–93. 10.1038/gt.2012.22 22378344

[43] Chromek M Arvidsson I Karpman D. The antimicrobial peptide cathelicidin protects mice from *Escherichia coli* 0157:H7-mediated disease. PLoS One (2012) 7:e46476. 10.1371/journal.pone.0046476 23077510 PMC3471911

[44] Loonen LMP Stolte EH Jaklofsky MTJ Meijerink M Dekker J van Baarlen P REG3γ-deficient mice have altered mucus distribution and increased mucosal inflammatory responses to the microbiota and enteric pathogens in the ileum. Mucosal Immunol (2014) 7:939–47. 10.1038/mi.2013.109 24345802

[45] Ricklin D Hajishengallis G Yang K Lambris JD. Complement: a key system for immune surveillance and homeostasis. Nat Immunol (2010) 11:785–97. 10.1038/ni.1923 20720586 PMC2924908

[46] Kemper C Atkinson JP Hourcade DE. Properdin: emerging roles of a pattern-recognition molecule. Annu Rev Immunol (2010) 28:131–55. 10.1146/annurev-immunol-030409-101250 19947883

[47] Lesher AM Nilsson B Song WC. Properdin in complement activation and tissue injury. Mol Immunol (2013) 56:191–8. 10.1016/j.molimm.2013.06.002 23816404 PMC3730815

[48] Spitzer D Mitchell LM Atkinson JP Hourcade DE. Properdin can initiate complement activation by binding specific target surfacs and providing a platform for *de novo* convertase assembly. J Immunol (2010) 179:2600–8. 10.4049/jimmunol.179.4.2600 17675523

[49] Selander B Martensson U Weintraub A Holmstrom E Matsushita M Theil S Mannan-binding lectin activates C3 and the alternative complement pathway without involvement of C2. J Clin Invest (2006) 116:1425–34. 10.1172/JCI25982 16670774 PMC1451204

[50] Tateishi K Matsushita M. Activation of the alternative complement pathway by mannose-binding lectin via a C2-bypass pathway. Microbiol Immunol (2011) 55:817–21. 10.1111/j.1348-0421.2011.00378.x 21831201

[51] Matsushita M Fujita T. Cleavage of the third component of complement (C3) by mannose-binding protein-associated serine protease (MASP) with subsequent activation. Immunobiology (1995) 194:443–8. 10.1016/S0171-2985(11)80110-5 8749236

[52] Takahashi M Ishida Y Iwaki D Kanno K Suzuki T Endo Y Essential role of mannose-binding lectin-associated serine protease-1 in activation of the complement factor D. J Exp Med (2010) 207:29–37. 10.1084/jem.20090633 20038603 PMC2812541

[53] Amara U Rittirsch D Flierl M Bruckner U Klos A Gebhard F Interaction between the coagulation and complement system. Adv Exp Med Biol (2008) 632:71–9. 19025115 10.1007/978-0-387-78952-1_6 PMC2713875

[54] Harboe M Mollnes TE. The alternative complement pathway revisited. J Cell Mol Med (2008) 12:1074–84. 10.1111/j.1582-4934.2008.00350.x 18419792 PMC3865650

[55] Lutz HU Jelezarova E Complement amplification revisited. Mol Immunol (2006) 43:2–12 10.1016/j.molimm.2005.06.020 16023211

[56] Halstensen TS Mollnes TE Garred P Fausa O Brandtzaeg P. Epithelial deposition of immunoglobulin G1 and activated complement (C3b and terminal complement complex) in ulcerative colitis. Gastroenterology (1990) 98:1264–71. 1691118 10.1016/0016-5085(90)90343-y

[57] Halstensen TS Mollnes TE Garred P Fausa O Brandtzaeg P. Surface epithelium related activation of complement differs in Crohn’s disease and ulcerative colitis. Gut (1992) 33:902–8. 10.1136/gut.33.7.902 1379568 PMC1379402

[58] Ahrenstedt O Knutson L Nilsson B Nilsson-Ekdahl K Odlind B Hallgren R. Enhanced local production of complement components in the small intestines of patients with Crohn’s disease. N Engl J Med (1990) 322:1345–9. 10.1056/NEJM199005103221903 2325733

[59] Riordan SM McIver CJ Wakefield D Andreopoulos PC Duncombe VM Bolin TD Local and systemic complement activity in small intestinal bacterial overgrowth. Dig Dis Sci (1997) 42:1128–36. 10.1023/A:1018821200354 9201072

[60] Andoh A Fujiyama Y Sumiyoshi K Bamba T. Local secretion of complement C3 in the exocrine pancreas: ductal epithelial cells as a possible biosynthetic site. Gastroenterology (1996) 110:1919–25. 10.1053/gast.1996.v110.pm8964419 8964419

[61] Laufer J Oren R Goldberg I Horowitz A Kopolovic J Chowers Y Cellular localization of complement C3 and C4 transcripts in intestinal specimens from patients with Crohn’s disease. Clin Exp Immunol (2000) 120:30–7. 10.1046/j.1365-2249.2000.01168.x 10759760 PMC1905612

[62] Sugihara T Kobori A Imaeda H Tsujikawa T Amagase K Takeuchi K The increased mucosal mRNA expressions of complement C3 and interleukin-17 in inflammatory bowel disease. Clin Exp Immunol (2010) 160:386–93. 10.1111/j.1365-2249.2010.04093.x 20089077 PMC2883109

[63] Ostvik AE Granlund AV Gustafsson BI Torp SH Espevik T Mollnes TE Mucosal toll-like receptor 3-dependent synthesis of complement factor B and systemic complement activation in inflammatory bowel disease. Inflamm Bowel Dis (2014) 20:995–1003. 10.1097/MIB.0000000000000035 24739633

[64] Müller S Schaffer T Flogerzi B Seibold-Schmid B Schnider J Takahashi K Mannan-binding lectin deficiency results in unusual antibody production and excessive experimental colitis in response to mannose-expressing mild gut pathogens. Gut (2010) 59:1493–500. 10.1136/gut.2010.208348 20682699

[65] Bernet-Camard MF Coconnier MH Hudault S Servin AL. Differential expression of complement proteins and regulatory decay accelerating factor in relation to differentiation of cultured human colon adenocarcinoma cell lines. Gut (1996) 38:248–53. 10.1136/gut.38.2.248 8801206 PMC1383032

[66] Andoh A Fujiyama Y Bamba T Hosoda S. Differential cytokine regulation of complement C3, C4, and factor B synthesis in human intestinal epithelial cell line, Caco-2. J Immunol (1993) 151:4239–47. 8409399

[67] Wirthmueller U Dewald B Thelen M Schäfer MKH Stover C Whaley K Properdin, a positive regulator of complement activation, is released from secondary granules of stimulated peripheral blood neutrophils. J Immunol (1997) 158:4444–51. 9127010

[68] Medof ME Walter EI Rutgers JL Knowles DM Nussenzweig V. Identification of the complement decay-accelerating factor (DAF) on epithelium and glandular cells and in body fluids. J Exp Med (1987) 165:848–64. 10.1084/jem.165.3.848 2434600 PMC2188295

[69] Cao Q McIsaac SM Stadnyk AW. Human colonic epithelial cells detect and respond to C5a via apically expressed C5aR through the ERK pathway. Am J Physiol Cell Physiol (2012) 302:C1731–40. 10.1152/ajpcell.00213.2011 22496247

[70] Chehoud C Rafail S Tyldsley AS Seykora JT Lambris JD Grice EA. Complement modulates the cutaneous microbiome and inflammatory milieu. Proc Natl Acad Sci U S A (2014) 110:15061–6. 10.1073/pnas.1307855110 23980152 PMC3773768

[71] Nordahl EA Rydengård V Nyberg P Nitsche DP Mörgelin M Mörgelin M Activation of the complement system generates antibacterial peptides. Proc Natl Acad Sci U S A (2004) 101:16879–84. 10.1073/pnas.0406678101 15550543 PMC534732

[72] Sonesson A Ringstad L Nordahl EA Malmsten M Mörgelin M Schmidtchen A. Antifungal activity of C3a and C3a-derived peptides against *Candida*. Biochim Biophys Acta (2007) 1768:346–53. 10.1016/j.bbamem.2006.10.017 17169328

[73] Lisewski MK Kolev M Le Friec G Leung M Bertram PG Fara AF Intracellular complement activation sustains T cell homeostasis and mediates effector differentiation. Immunity (2013) 39:1143–57. 10.1016/j.immuni.2013.10.018 24315997 PMC3865363

[74] Leffler J Martin M Gullstrand B Tydén H Lood C Treudsson L Neutrophil extracellular traps that are not degraded in systemic lupus erythematosus activate complement exacerbating the disease. J Immunol (2012) 188:3522–31. 10.4049/jimmunol.1102404 22345666

[75] Bhat S Song Y-H Lawyer C Milner SM Modulation of the complement system by human β-defensin 2. J Burns Wounds (2006) 5:e10. 17235375 PMC1769518

[76] Liu Y Liu Y Zhang X Wang J Li Z Pang X Complement-related proteins control the *Flavivirus* infection of *Aedes aegypti* by inducing antimicrobial peptides. PLoS Pathog (2014) 10:e1004027. 10.1371/journal.ppat.1004027 24722701 PMC3983052

